# Cost of Church and Theatre

**Published:** 1857-05

**Authors:** 


### COST OF CHURCH AND THEATRE.

CALCULATIONS and estimates have been made and published as to the amount which it costs individual church members to sustain their respective churches, and in such a way as to produce the impression that it is inconsistent with the sentiment that the gospel should be free to all. A great many of our secular papers do not hesitate to give a side-strike at religion, when they can do it by an innuendo.

| | |
|---|---|
| Cost of a Baptist, per annum, . . | $3 40 |
| " Methodist, . . . . | 3 40 |
| " Presbyterian, . . . | 7 00 |
| " Congregationalist, . . . | 10 00 |
| " Roman Catholic, . . . | 14 00 |
| " Episcopalian, . . . | 18 00 |
| " Reformed Dutch, . . . | 22 00 |
| " Unitarian, . . . . | 23 00 |

Supposing these estimates are correct, there is one thing to be kept in view: the money thus furnished belonged to the donors, and if they saw proper to spend it in securing for themselves and families the opportunities of religious worship, we do not see exactly why it should be invidiously remarked upon, especially as the largest portion of all the charities of the land are sustained by those who habitually attend church on Sundays. Those are they who give largely while living, and as largely when they die. Aye, millions do they give every year, to teach the blind, to house the homeless, to clothe the naked, to feed the hungry, to nurse the sick, to reclaim the abandoned, and to save the orphan from idleness, and crime, and disease—thus alleviating the burdens of to-day, and preventing their occurrence on to-morrow. There is nothing in church-going to entail poverty, disease, destitution, and crime.

Suppose we make some calculations as to what it costs to support another institution—the drama, the theatre. Not less than twenty thousand dollars are expended every twenty-four hours in New York city, in connection with the various branches of theatrical performances, including tickets, carriage hire, refreshments, brandy, gin, whiskey, lager-bier, and worse things still. Seven millions of dollars a year in New York alone are expended in connection with theatrical performances. Let it be so that the money thus expended belongs legitimately to the persons who thus spend it, which nobody who knows the character of the mass of habitual theatre-goers will for a moment contend. Look at the grog shops, and drinking saloons, and gambling establishments which swarm around the theatre. A theatre without a house for drunkenness and prostitution within hailing distance, would be out of its element; it would die of inanition. We know of no theatre whose boards are guiltless of the frequent sneer, and slur, and bitter sarcasm as to religion and its ministers.

If the drama is so elevating in its tendencies, it is rather difficult accounting for the fact that not only those who patronize it do at the same time sustain the bar, the billiard-saloon, and the assignation house, but even those who dispense the drama, the actors and actresses, patronize these same things, with chance exceptions; and in dying, in vain do we look for those benevolences which build asylums, found colleges, and sustain hospitals—which provide for the aged widow, and give the orphan child a home. Moses Shepherd dies and gives half a million to secure a home and a keeper for the unfortunate lunatic and the harmless idiot. Another, who lived by the drama, and made by it a fortune, gave it to another in his own line of business, while his aged mother was left to starve, and his own brothers and sisters were cut off without a shilling.

With these divers calculations, we ask which is the best investment; which pays the largest dividend to humanity—the church or the theatre?—the church, with its quiet Sabbaths, its nights for repose, its countless academies, and colleges, and seminaries, its hospitals, its asylums, its "retreats"—or the theatre, with its midnight saturnalia at the eating saloon, the dram shop, the billiard room, the pimp house; crowding the lock-ups, filling the jails, peopling the penitentiaries, or occupying with its patrons and its victims the very hospitals and asylums which church-goers have builded and sustain!

